# Editorial: Advances, opportunities and challenges of using modern AGI and AIGC technologies in depression and related disorders

**DOI:** 10.3389/fpsyt.2025.1625579

**Published:** 2025-06-04

**Authors:** Ming D. Li, Yunpeng Wang

**Affiliations:** ^1^ State Key Laboratory for Diagnosis and Treatment of Infectious Diseases, National Clinical Research Center for Infectious Diseases, Collaborative Innovation Center for Diagnosis and Treatment of Infectious Diseases, The First Affiliated Hospital, Zhejiang University School of Medicine, Hangzhou, China; ^2^ Department of Psychology, University of Oslo, Oslo, Norway

**Keywords:** artificial intelligence, digital medicine, psychiatry, ChatGPT, big-data, foundation model, depression

OpenAI has recently released GPT-4 (or called ChatGPT plus), which represents one small step for generative AI (GAI), but one giant step for artificial general intelligence (AGI). The capabilities of ChatGPT in the medical field have been already assessed in several works, such as cirrhosis and hepatocellular carcinoma (HCC) diagnosis and prevention, and answering frequently asked questions about diabetes. In a study conducted to identify appropriate imaging for patients requiring breast cancer screening and assessment for breast pain, it was exciting to find that ChatGPT achieved an accuracy of 56.25% even with the worst-performing set of metrics, when assessing whether the proposed imaging modality complies with the American College of Radiology (ACR) guidelines.

As to the psychiatric field, current ChatGPT uses have been mainly limited to assisting psychiatrists with clinical routine tasks, while tasks of screening and diagnosis, psychotherapy, or patient assessments are still commonly performed by trained therapists. Although various trials of ChatGPT-assisted mental health services have been pursued, such as ChatBeacon, an all-inclusive customer service platform providing mental health assistance powered by ChatGPT; and Koko, a free therapy program testing a demo of GPT-3 mental health intervention, current discussion about the potential uses of AGI in psychiatry is still quite limited. As AGI, especially GPT-like technologies, gains more power and efficiency, we can foresee that there will be increasing directions to further integrate and expand AGI applications in clinical psychiatry in the near future.

For example, ChatGPT may help deal with the burnout issue encountered by psychiatrists in psychiatric clinics, by assisting processing transcripts of clinical dictations to generate summaries from medical dialogues and then entering into medical records as admission notes. ChatGPT can also help complete medical record documentation in standard or customized formats to reduce stressful bureaucracy and protect mental health professionals from burnout. On the other hand, ChatGPT may also facilitate the communication process between psychiatrists and other doctors and healthcare professionals by providing templates or polishing the contents in consultation letters, notes, and other clinical communications; as well as communication between patients and psychiatrists by generating appropriate patient clinic letters with humanity.

Despite continuous advances in the GPT model, we must consider significant ethical challenges for widespread application in psychiatry and healthcare. For example, advanced GPT-4 model still has potential risks of providing harmful advices due to the fluid nature of language and associated training data sets. Therefore, before commercial release, extensive training, adjustments, and comprehensive evaluation of a fully-automated psychotherapy system should be conducted to minimize the risk of harm to patients. Furthermore, it should be mandatory and necessary for mental health professionals to adopt a safer supervision operational mode when using AI systems as assistants, through routine monitoring of patients and identifying the erroneous performance of AI systems. Like many other past technological advances introduced in health care, GPT technology is envisioned to enhance the professional team’s capabilities to deliver more efficient and effective care only after much validation and real-world testing. Therefore, it is essential for researchers to re-examine issues such as professional and ethical standards in patient-physician relationships, and to construct new diagnostic, therapeutic, and training models that incorporate digital health care into clinical practice.

Under this scenario, this Research Topic aims to provide an updated status of AI in the field of psychiatry and envision a potential future of digital mental health care *via* integration and advances in AGI technology and its supporting devices such as knowledge tree and hardware, etc. Of the five papers included in this Research topic, three are reviews focusing on a historical review of AI development in psychiatry (Liu et al.), a proposed new framework on digital psychiatry (Wu and Li, 2025, under revision), and the development of knowledge graph and its application on psychiatry (Wang et al.) and two are original research reports with the first one describing a model approach that can handle multidimensional psychiatric data (Zhang et al.) and the other on VR development and its application in psychiatry (Wang et al.).

The first paper in the current Research Topic was reported by Liu et al., which provides a systematic review of the research progresses of integrating AI technology with depression diagnosis (Liu et al.). The authors first describe the knowledge-driven first-generation of depression diagnosis methods, which could only address deterministic issues in structured information with the selection of depression-related features directly influencing identification outcomes. The data-driven second-generation of depression diagnosis methods achieved automatic learning of features but required substantial high-quality clinical data, and the results were often obtained solely from the black-box models which lack sufficient explainability. In an effort to overcome the limitations of the preceding approaches, the third-generation of depression diagnosis methods combined the strengths of knowledge-driven and data-driven approaches. Through the fusion of information, the diagnostic accuracy is greatly enhanced, but the interpretability remains relatively weak. In order to enhance interpretability and introduce diagnostic criteria, this paper offers a new approach using Large Language Models (LLMs) as AI agents for assisting the depression diagnosis. Finally, the authors also discuss the potential advantages and challenges associated with this approach. This newly proposed innovative approach has the potential to offer new perspectives and solutions in the diagnosis of depression.

The second review featured in the current Research Topic dealt with the newly proposed concept of digital psychiatry which is being considered as a specialty to focus on combining the psychiatric clinical practices, psychiatric knowledge, and modern intelligent/digital approaches to automate the psychiatric clinical processes, such as diagnosis and treatment, in order to yield faster, better and consistent results (Wu and Li, 2025). On the basis of recent advances in large-scale pre-trained models (PTMs), digital humans, virtual reality (VR) and other immersive techniques, the authors propose a framework to fully-automate the processes of mental health practices, and thus pave the way for digital psychiatric clinics. Specifically, in this review, the authors first provided an outline of the related technological advances to digital mental health care, by detailing how digital entity, medical domain knowledge, autonomous agents and virtual reality present new opportunities for practical clinical uses. Then, the authors introduce several basic mental health clinical topics used in real-world settings and propose the framework of developing a fully-automated digital psychiatric system building on the existing artificial intelligence (AI) related technologies. Finally, they discuss the challenges of implementing the digital psychiatry in the real-world environments.

The third study in this Research Topic focused on the application of knowledge graph (KG) technique in data management and data utilization in the study of neurological and mental disorders (Wang et al.). As research on human medical disorders advances, so does the body of medical data. The accumulation of such data provides unique opportunities for the basic and clinical study of these diseases, but the vast and diverse nature of the data also make it difficult for physicians and researchers to precisely extract the information and utilize it in their work. KG, as an organized form of information, has great potential for the study neurological and mental disorders when it is paired with big data and deep learning technologies. In this paper, the authors first reviewed the application of KGs in common neurological and mental disorders in recent years. Then they discussed the current state of medical knowledge graphs, highlighting the obstacles and constraints that still need to be overcome.

The fourth study of this Research Topic dealt with the introduction of a Sentence-level Multi-modal Feature Learning (SMFL) approach, which can analyze synchronized sentence-level segments of facial expressions, vocal features, and transcribed texts obtained from patient-doctor interactions (Zhang et al.). The SMFL approach incorporates Temporal Convolutional Networks (TCN) and Long Short-Term Memory (LSTM) networks to meticulously extract features from each modality, aligned with the structured temporal flow of dialogues. By analyzing the collected multi-modal features from 100 participants (67 depressed patients and 33 non-depressed individuals and a public available DAIC-WOZ with 192 participant data included, the proposed SMFL achieved an accuracy of 91% and 89%, respectively, demonstrating superior performance in binary depression classification. This advanced approach not only achieves a higher precision in identifying depression but also ensures a balanced and unified multi-modal feature representation, demonstrating superior performance in binary depression classification. However, due to the concern of small sample size in this study, the value and applicability of the proposed approach requires further research in large samples.

The last study included in this Research Topic, by Wang et al., focused on the virtual reality (VR) technology and its combination with cognitive behavioral therapy (VR-CBT), which has emerged as a promising treatment for diagnosing and treating mental illnesses such as anxiety, depression, and attention deficit hyperactivity disorder (ADHD) (Wang et al.). By analyzing reported researches on the application of VR in anxiety, depression, and ADHD, the authors concluded that VR is an effective tool for supporting the treatment of mental illnesses across various settings and recommend its incorporation into clinical practice.

